# Comparison of two 3D scanning software to identify facial features: a prospective instrument to predict difficult airway

**DOI:** 10.1186/s13741-024-00362-2

**Published:** 2024-02-22

**Authors:** Alexander Rocuts, Bibiana Avella-Molano, Amanda Behr, Farhan Lakhani, Bryant Bolds, Matias Riveros-Amado, Efrain Riveros-Perez

**Affiliations:** 1 Department of Anesthesiology and Perioperative Medicine, Medical College of Georgia at Augusta University, Augusta, GA USA; 2 CAHS - Department of Medical Illustration, Augusta University, Augusta, GA USA; 3 Lakeside High School, Evans, GA USA; 4 Department of Anesthesiology and Perioperative Medicine, Outcomes Research Consortium, Cleveland Clinic, Medical College of Georgia at Augusta University, Augusta, GA USA

**Keywords:** Three-dimensional imaging, Anesthesiology, Airway management

## Abstract

**Background:**

Clinical airway assessment has limited predictive ability to anticipate difficult airway. Three-dimensional (3D) technologies have emerged in medicine as valuable tools in different settings including innovation and surgical planning. Three-dimensional facial scanning could add value to clinical measurements and two-dimensional models to assess the airway. However, commonly used high-fidelity scans are expensive. This study aims to compare the accuracy of the measurements made by the Scandy Pro app as a cost-effective alternative to high-fidelity scans made by the Artec Space Spider. We also aim to evaluate the interobserver variability for the measurements performed with Scandy Pro.

**Materials and methods:**

We conducted a cross-sectional, comparison study on 10 healthy volunteers. Four observers measured 720 distances and 400 using both Scandy Pro and Artec Space Spider facial scans. Wilcoxon test was used for group–group comparison.

**Results:**

Comparison of both instruments showed no difference in angle or distance measurements. The percentage error (measurement difference between the two devices) exhibited by one of the observers was significantly different compared with the other three observers; however, the magnitude of this individual deviation did not affect the overall percentage error. The overall error for Scandy Pro was 5.5% (3.9% and 6.7% for angles and distances, respectively).

**Conclusion:**

Three-dimensional facial scanning with Scandy Pro is an accurate tool that can be a cost-effective alternative to high-fidelity scans produced by the Artec Space Spider.

## Background

Mask ventilation and endotracheal intubation are critical steps in airway management in both elective and emergency settings. The correct identification of potential difficult to mask-ventilate/intubate patients is essential to avoid adverse and potentially lethal outcomes (Vannucci and Cavallone 2015; Mahmoodpoor et al. 2017; Law et al. 2021; Joffe et al. 2019). Several predictors have been proposed to identify difficult airway. Some of these predictors have exhibited suboptimal performance when used alone. Combination of different indexes yields better diagnostic accuracy; however, an index predictor with consistent clinically reliable performance in terms of specificity and sensitivity is still lacking (Vannucci and Cavallone 2015; Joffe et al. 2019; Shah and Sundaram 2012; Adi et al. 2021). Hence, clinicians from different disciplines are embarking in a continuous search for newer and better predictors for airway difficulty assessment (Adi et al. 2021; Agarwal et al. 2021).

Photogrammetry is the use of photography to measure distances between points on a three-dimensional (3D) surface (Slaker and Mohamed 2017). It has been used in medicine mainly for surgical planning, and it might be a valuable tool for airway assessment (Singh and Singh 2021). One limitation of 3D scanning for this purpose may be the high cost of the instruments used for photogrammetry (Siapno et al. 2020). The Artec Space Spider retails at US $24,800, whereas the Scandy Pro app is available as either a US $5.99 per month or US $39.99 per year subscription (Europe A 2023). Newer commercially available technologies use similar 3D computer-generated mesh models for facial recognition. These software options offer enhanced usability in the perioperative setting by allowing clinicians to conveniently access the tool using a portable iPhone, eliminating the need for an additional larger, device to be carried. As facial recognition software becomes more accessible, there is enormous potential for it to be integrated in the armamentarium of difficult airway evaluation tools. Our primary aim is to assess the accuracy of the distance and angle measurements made by the commercially available smartphone application Scandy Pro app (Scandy Pro 1.9.10, Scandy LLC, New Orleans, LA, USA) compared to the high-fidelity portable scanning device: Artec Space Spider (Space Spider, Artec 3D, Santa Clara, CA, USA). Our secondary aim is to evaluate the interobserver variability for the measurements performed with Scandy Pro.

## Materials and methods

This study was reported according to the SQUIRE 2.0 guidelines (Ogrinc et al. 2015). After the institutional review board approval (protocol no. 1845163), a cross-sectional study was conducted to assess the accuracy of the 3D facial scan measurements performed with a commercially available software device. An honest broker recruited medical students through direct contact to determine their willingness to participate in our study. After a detailed description of the study, each participant gave written signed consent for their participation in the study. A total of 10 medical students were recruited. A triangle was drawn on one of the cheeks of each participant, and afterward, a 3D facial scanning was performed with a high-fidelity scan (Artec Space Spider) and repeated with an iPhone app (Scandy Pro). The 3D facial scanning done with the high-fidelity scans was exported in an OBJ and JPG format, and the ones performed in the iPhone app were exported in a PLY format to analyze the facial scanning with color in an open-access 3D computer graphics software (Blender) in order to perform the measurements. Utilizing the same software to measure obtained data from 3D face scans allowed us to isolate changes in measurements to the scanning device. We opted to use an open-access 3D software due to its accessibility.

### 3D facial scanning devices

The Artec Space Spider 3D scanner was used as the benchmark scanner because of its accuracy and reputation in the medical and engineering industries. Numerous studies have tested its precision, and the results indicate that it is comparable to that of a measuring tape and a goniometer (Winkler and Gkantidis 2020; Walker et al. 2022). Furthermore, it has been demonstrated to be a precise device with an average deviation of less than 1 mm (Hollander et al. 2021; Probst et al. 2022). Overall, the Artec Space Spider 3D scanner is a scientifically approved and reliable tool for capturing accurate 3D scans.

The Spider emits blue LED light scanning technology to convert target objects into 3D meshes inside the computer. Blue light scanning is a highly accurate scanning technology where the scanner produces a blue LED light that bounces off the scanned object. A camera on the scanner captures the light bouncing back from the object, and the scanner creates a 3D calculation of the object’s position and shape. The scanner captures surface color through a six LED light white LED flash. The scanner has up to 0.05-mm 3D accuracy and up to 0.1-mm 3D resolution (Europe A 2023).

The Scandy Pro app, available at an affordable price on the Apple App Store, takes advantage of the depth map created by Apple’s TrueDepth sensor found on the front-facing camera of recent iPhone models (iPhone X and later). Scandy Pro meshes the points obtained to capture a 3D scan of an object. Regarding the Apple TrueDepth sensor, this sensor works by using an infrared emitter projecting over 30,000 dots which are photographed by an IR camera and subsequently analyzed for depth mapping. In low light conditions, the Apple TrueDepth sensor can utilize a flood illuminator to add light to the subject. The scan captures per-vertex color, which means a texture map is not generated. However, the per-vertex color may be surfaced with a PLY file, which was performed in the study to obtain measurements that did not rely on significant depth changes, for example, points corresponding to the triangle drawn on participants’ faces. Scandy Pro states that they do not store any ARKit, Apple’s augmented reality platform for developers, information and do not share any ARKit information with anyone.

We operated at the furthest distance where Scandy Pro could acquire data at the highest resolution offered in the app. We applied a similar procedure to the Artec Space Spider scans where the device was operated at the furthest distance that could generate the 3D scan.

### Measurements

**Fig. 1:**
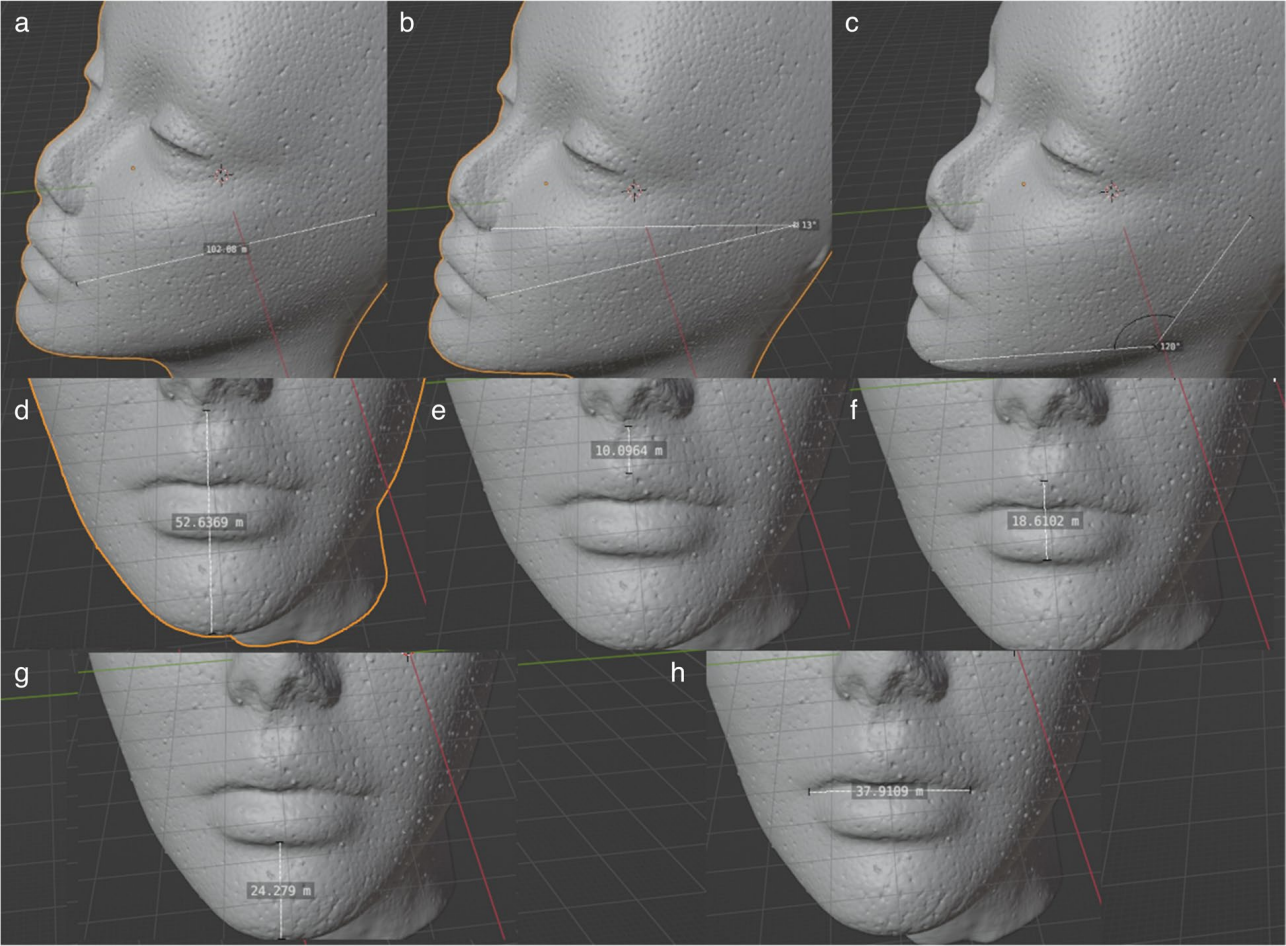
Manikin head illustrating the measurements performed on each facial scan. **a** Ear tragus to lip corner distance. **b** Nose-tragus-lip. **c** Mandible angle. **d** Nose bottom to chin distance. **e** Nose bottom to upper lip distance. **f** Upper to lower lip distance. **g** Lower lip to chin distance. **h** Lip horizontal length

**Fig. 2:**
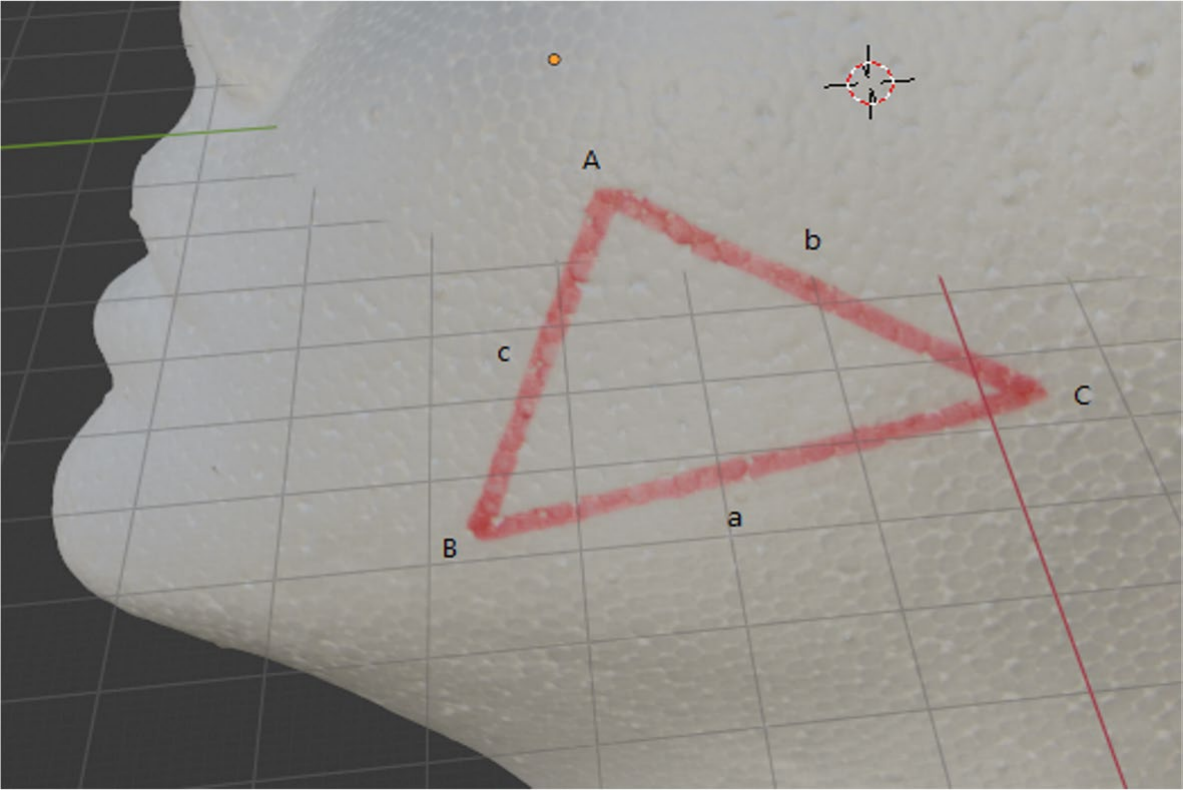
Triangle. A Top angle. a Bottom distance. B Bottom angle. b Top distance. C Third angle. c Third distance

A total of nine distances and five angle measurements were chosen to be measured on each medical students’ facial scans. Afterward, a 3D facial scanning was performed with each of the devices. Four observers were instructed through a 30-min presentation on how to perform the measurements on each facial scan using an open-access 3D computer graphics software (blender). Distances and angles that correlated with mouth opening and jaw angle were selected based upon their ability to predict a difficult airway (Mahmoodpoor et al. 2017; Shah and Sundaram 2012; Amornvit and Sanohkan 2019; Rudy et al. 2020; Suzuki et al. 2007; Naguib et al. 1999; Connor and Segal 2011). The triangle measures were selected to provide predictive comparisons between the accuracy of each tool. To evaluate the interobserver variability in the measurements, four observers performed the measurements in each scan. Figures 1 and 2 illustrate the measurements performed in each face scan.

### Statistical analysis

All statistical analyses were performed using JMP® Version 16.0.0 (SAS Institute Inc. Cary, NC, USA). The distribution of our three response variables (length, angles, and percent error) was evaluated with a histogram. After assessing normality of the observations with normal quantile plot and Shapiro–Wilk test, we concluded that our three response variables’ distributions deviate from normal distribution. Specifically, the length and percent error displayed a left-skewed distribution, while the angles had a right-skewed distribution. Therefore, after conducting a Levene test to ensure equal variances, we utilize the Wilcoxon test, which is a nonparametric test, to assess if there are any significant differences between groups. Utilizing the Wilcoxon test, which is a nonparametric test, we compared the accuracy in measurements obtained from the Scandy Pro app versus the high-fidelity scans. Additionally, we used the Wilcoxon test to assess the interobserver variability by comparing the percentage error between observers. Finally, a Bland–Altman plot was used to depict if there is an agreement between the two methods used to perform the facial scans.

For this study, we utilized convenience sampling and then carried out a post hoc power analysis. To compare two independent sample groups, we opted for the nonparametric Wilcoxon test and employed the Guenther method. Based on an effect size of 1 mm, our analysis revealed a power of 70%.

## Results

The distance and angle measurements were performed on the facial scans of 10 healthy volunteers. A total of 720 distances and 400 angles were collected. Half of the measurements were made on Artec Space Spider facial scans and the other half on Scandy Pro facial scans. Distance and angle measurements deviate from normal distribution as evidenced by the normal quantile plot and a statistically significant Shapiro–Wilk test (< 0.001). Therefore, nonparametric tests were performed to compare the groups.

The Wilcoxon test showed no statistically significant difference between both instruments’ median angle and distance measurements. This finding was present when analyzing the overall angle measurements and the overall distance measurements as well as each of the measurements taken separately (Table 1).

**Table 1:** Descriptive analysis and Wilcoxon test for distance (cm) and angle (degrees) measurements

Measurement	*n*	Scandy pro	Artec Space Spider	*p*-value
		Median (IQR)	Median (IQR)	
Ear tragus to lip corner	80	10.4 (1.17)	10.2 (1)	0.7983
Nose bottom to chin	80	6.35 (0.7)	6.4 (0.9)	0.6397
Nose bottom to upper lip	80	1.4 (0.5)	1.4 (0.3)	0.4230
Upper to lower lip	80	1.6 (0.8)	1.55 (0.78)	0.1259
Lower lip to chin	80	3.25 (0.9)	3.25 (0.78)	0.8207
Lip horizontal length	80	4.8 (0.67)	4.8 (0.58)	0.4603
Top triangle distance	80	5.3 (1.12)	5.1 (1.5)	0.6232
Bottom triangle distance	80	5.25 (1.45)	5.3 (1.05)	0.4730
Third triangle distance	80	4.5 (1.15)	4.45 (1.38)	0.6130
Total distances	720	4.7 (3.1)	4.7 (3.1)	0.8283
Nose-tragus-chin angle	80	32 (4.5)	31 (7)	0.6326
Mandible angle	80	125 (4)	124 (6.75)	0.2668
Top triangle angle	80	66 (16.25)	65 (17.75)	0.6130
Bottom triangle angle	80	64.5 (14)	64.5 (18)	0.8889
Third triangle angle	80	48 (8)	47.5 (8.75)	0.9885
Total angles	400	61.5 (34)	61 (34)	0.7989

*IQR* Interquartile range, *n* sample size

**Fig. 3:**
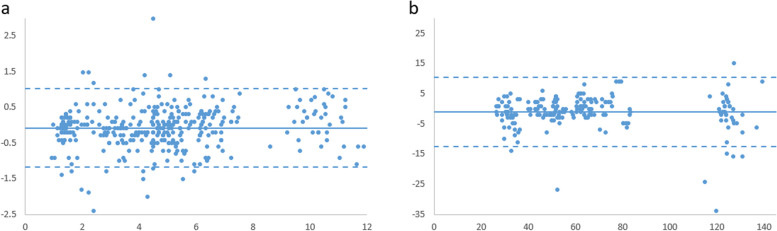
Bland–Altman plot for **a** lengths and **b** angles

According to the Bland–Altman plot in Fig. 3, the mean difference bias is close to zero for length and angle measurements. Most data points are clustered around the mean difference and fall within the limits of agreement, especially for the length measurements. These findings suggest that the measurements obtained with both devices are in agreement.

Percentage error for each of the measurements was calculated using the Artec Space Spider facial scans as the standard. The percent error for the overall sample size was 5.5% (Table 2). When comparing the median percentage error between the four different observers, a statistically significant difference was found for the overall group of measurements (*p* = 0.0198) and the distance measurements (*p* = 0.0282) but not for the angle measurements (*p* = 0.4269).

**Table 2:** Percentage error (%) and Wilcoxon test *p*-value

	Percentage error with all the observers	Percentage error without observer 1	*p*-value
	Median (IQR)	Median (IQR)	
Angles	3.9 (5.4)	3.7 (5)	0.5699
Distances	6.7 (9.5)	6.3 (8.7)	0.2633
Total sample	5.5 (8.7)	5.13 (7.8)	0.2472

*IQR* Interquartile range

When performing the nonparametric comparisons for each pair using Wilcoxon method, a statistically significant difference in the median percentage error was evidenced between one of the observers against the others (Table 3). We evaluated if this difference impacted the overall percentage error. The Wilcoxon test showed no statistically significant difference in the percentage error independently of the inclusion of observer 1 (Table 2).

**Table 3:** Nonparametric comparisons for each pair using Wilcoxon method for percent error

	Observer 1	Observer 2	Observer 3
Observer 1	**-**	-	**-**
Observer 2	0.0042^a^	-	-
Observer 3	0.0207^a^	0.6195	-
Observer 4	0.0207^a^	0.4679	0.8570

*p*-values for the nonparametric comparisons for each pair using Wilcoxon method for percent error between observers

^a^Statistically significant

## Discussion

Our study did not evidence statistically significant differences in the angle or distance measured on facial scans obtained from either of the instruments used (Scandy Pro and Artec Space Spider). The percentage error for the Scandy Pro was 5.5% (3.9% and 6.7% for angles and distances, respectively). There was a statistically significant difference in percentage error of one of the observers compared to the other three; however, no statistically significant difference was demonstrated in the percentage error with or without the observer 1 measurements.

In recent years, the use of three-dimensional scanning has generated enthusiasm in the medical field, especially in the surgical setting, where its applications range from device innovation to surgical navigation (Zahia et al. 2022; Amornvit and Sanohkan 2019). The scope of such applications is expanding to other medical specialties. High cost is a limitation to the widespread use of 3D scanning. We propose the use of Scandy Pro as a cost-effective alternative to high-fidelity 3D scanning devices (Rudy et al. 2020; Bartella et al. 2022). Our results demonstrate that an affordable option like Scandy Pro has satisfactory accuracy for angle and distance measurement. Furthermore, the universal use of smartphones makes it a very accessible tool.

In the field of anesthesiology, photography and other two-dimensional (2D) images have been used in the past to generate predictive models for difficult airway (Suzuki et al. 2007; Naguib et al. 1999; Cuendet et al. 2015; Langeron et al. 2012). Connor et al. used three-dimensional image reconstruction from 2D photographs (Connor and Segal 2011). To our knowledge, there are no published reports of 3D facial scanning to characterize facial features in order to develop a predictive model. We consider that 3D facial scanning possesses an advantage over 2D images such as photography and computerized tomography as it can optimize the accuracy of the measurements and predictions. By adding the third dimension, 3D facial scanning exhibits closer proximity to the real facial structure. Additionally, it eliminates the risk of radiation associated with computerized tomography.

We propose here the use of 3D scanning technologies to the evaluation of the airway. In our study, we compare the percentage error between different observers to determine the existence of interobserver variability. Although one of the observers’ percentage errors differed from the others, this difference did not affect the percentage error of the total sample. We consider that interobserver variation can be minimized with a more detailed standardization of the measurements. Another consideration of using commercially available 3D scanning is the potential for privacy or Health Insurance Portability and Accountability Act (HIPAA) violations, as 3D facial scans contain detailed biometric information. The imaging data obtained from these devices should be handled with the same rigor applied to other electronic personal health information (e-PHI) systems, abiding by the national standards of HIPAA to maintain patient privacy and security (Parts 2003). In addition to the strict use of password-protected policies, smartphone built-in encryption and remote data erasure may prove to be important tools to enhance HIPAA compliance (Leydon and Schwartz 2020).

Previous studies using clinical predictors and 2D imaging have demonstrated that using difficult airway predictors in conjunction rather than in an isolation enhances predictive performance (Naguib et al. 1999; Connor and Segal 2011; El-Radaideh et al. 2020). Future research should focus on the use of facial proportions validated in previous 2D models as a foundation to build 3D-scan predictive models in hopes to increase clinically relevant prediction of difficult airway. We consider that utilizing 3D scans could be a superior alternative to current bedside physical exams and 2D images, given that it has been demonstrated to be an accurate and precise tool that reduces interobserver variability (Walker et al. 2022; Hollander et al. 2021; Probst et al. 2022).

Our study had several limitations. First, we included only four observers, which might have limited our ability to evaluate interobserver variability. Furthermore, due to the presence of soft tissue surrounding the angle of the mandible, it was not easy to accurately identify bony landmarks. This limitation could be mitigated by color marking the angle of the mandible. Our participants, although inclusive of a range of skin tones, may not have been reflective of global or US populations. In addition, the external validity of our study is affected by the exclusion of bearded patients, which constitute an important subpopulation at risk of presenting with unanticipated difficult airway. Furthermore, Scandy Pro is not available for android devices; however, if future research demonstrates it has a good performance for difficult airway prediction, it is possible for different operating systems to support it. Another, one potential drawback of using the Scandy Pro app in a clinical setting is that the scans need to be processed offline to gather measurements before being entered into an algorithm for prediction. Finally, based on our calculated post hoc power of 70%, we should consider the risk of type 2 error in our study. This risk can be avoided in future studies by increasing the sample size.

## Conclusion

Three-dimensional facial scanning with Scandy Pro is an accurate tool that can be a cost-effective alternative to high-fidelity scans.

## Data Availability

The datasets used and/or analyzed during the current study are available from the corresponding author on reasonable request.
